# Editorial: Psychological Distress, Burnout, Quality of Life, and Wellness Among Healthcare Workers

**DOI:** 10.3389/fpsyg.2022.913941

**Published:** 2022-04-29

**Authors:** Laura Galiana, Krystyna Kowalczuk, Noemí Sansó

**Affiliations:** ^1^Department of Methodology for the Behavioral Sciences, University of Valencia, Valencia, Spain; ^2^Department of Integrated Medical Care, Medical University of Bialystok, Bialystok, Poland; ^3^Department of Nursing and Physiotherapy, University of the Balearic Islands, Palma, Spain; ^4^Balearic Islands Health Research Institute (IDISBA), Palma, Spain

**Keywords:** distress, burnout, quality of life, wellbeing, occupational health, organizational behavior, healthcare professionals, psychological intervention

The COVID-19 pandemic has increased pressure on health systems, forcing staff to make critical decisions in environments with multiple adverse conditions (Lluch et al., 2022). Certainly, health professionals are under extreme psychological pressure and, consequently, are at risk of developing several psychological symptoms and mental health disorders (Rajkumar, 2020), including anxiety, depression, stress, somatic symptoms, helplessness or loneliness (Barello et al., 2020; Galli et al., 2020; Kotera et al., 2021) Thus, this health crisis is bringing visibility to a problem that already existed. Understanding those factors that improve professionals' wellbeing, as well as the quality of care they provide, is key for adequate healthcare institutions functioning. Indeed, literature has started to link better health and wellbeing of healthcare professionals with better quality of care of patients. This Research Topic collects 13 research articles that develop our understanding of the psychological wellbeing and quality of life of healthcare workers, together with those coping strategies and organizational factors that can improve it, while reducing psychological distress and burnout.

Many of the works gathered in this Research Topic have focused on anxiety and depression of healthcare professionals and students. For example, Yang et al. studied clinical frontline medical staff from Chinese hospitals. These authors found that, in the later stages of the pandemic in China, the occurrence rates of anxiety and depression among medical staff remained high. Among the related variables, age, the need for psychological counseling, and the coexistence of depression were significantly associated with anxiety. Coexisting anxiety was also associated with the occurrence of depression. Peng et al., also in Chinese healthcare workers, analyzed the factors that influenced their mental health status. They explored the relationships of overcommitment, perceived troubles at work, perceived tension, and the capability to persist for >1 month at the current work intensity with anxiety and depression. Results of the Bayesian networks analysis found that the last three factors directly affected anxiety and depression. Other factors associated with anxiety and depression symptoms in Chinese healthcare workers have been suspicious symptoms on their own and in family members, working on the frontline, quarantine, and negative coping style, as reported by Wang et al. In this same line, intensive working schedule and high-risk environment, compounded by unfamiliar work setting and colleagues, local culture adaptation and isolation from usual social circle, were pointed by Khoo et al. as strainers of Chinese deployed healthcare workers. Meanwhile, reciprocal relationships and family relatedness with patients and colleagues, together with organizational support, formed crucial wellbeing resources in sustaining these professionals.

Also regarding anxiety and depression, but this time in Ecuadorian healthcare students and early-career professionals, Bonilla-Sierra et al. found that inflexibility and loneliness were important predictors of anxiety and depressive mood, specifically by mediating the relation between stress and this symptomatology. Additionally, in an attempt to improve our understanding of the psychological distress experienced by healthcare professionals during the pandemic, Ruiz-Frutos et al. studied predictors of mental health in a sample of Spanish healthcare professionals. These authors found that workload, conflicts, stressful situations, and less job satisfaction and work engagement were significantly related to higher psychological distress.

In Polish nurses, Larysz and Uchmanowicz examined the associations of sociodemographic characteristics and depression. The results showed that higher level of education, more job seniority, and living in a relationship were associated to lower levels of depression. Also in Polish nurses, Uchmanowicz et al. and Kołtuniuk et al. studied rationing of nursing care. This rationing has been reported worldwide, and implies nurses being forced to selectively perform care tasks, while omitting or delaying specific nursing tasks. As such, rationing of nursing care poses a direct threat to patient safety. Uchmanowicz et al. found a positive, statistically significant relation between burnout and rationing of nursing care. Meanwhile, Kołtuniuk et al. found that the patient-to-nurse ratio and the level of job satisfaction were also significant predictors of rationing of nursing care. Thus, these studies made important contributions to the underlying mechanisms of rationing of nursing care, highlighting the importance of burnout, patient-to-nurse ratio, and the level of job satisfaction for improving patient safety.

Important work has also been compiled regarding compassion fatigue of healthcare professionals. Ruiz Fernández et al., for example, studied the role of emotional intelligence and perceived health in relation to compassion fatigue in Spanish nurses. These authors found that the three components of emotional intelligence (attention, clarity, and repair), along with perceived health, influenced compassion fatigue. Nurses with higher levels of compassion fatigue used emotional care as a mechanism of emotional management and had poorer perceived health. Serrão et al. also focused on compassion fatigue, together with compassion satisfaction, this time in Portuguese physicians and nurses. Their results pointed that most of the participants showed moderate levels of compassion fatigue, in its two dimensions: secondary traumatic stress and burnout. Evidence pointed that female gender was significantly associated with more susceptibility to secondary traumatization, whereas factors that contributed to burnout included years of professional experience and the number of work hours per week.

Finally, two more key issues involved in healthcare professionals' wellness were studied in this Research Topic. Schneider et al. studied moral distress in German healthcare workers, with findings pointing that moral distress was strongly correlated with depressive symptoms, anxiety symptoms, occupancy rate at current work section, and contact with COVID-19. They also found that moral distress was higher for nurses and medical technical assistants compared to other groups, such as physicians or psychologists. Therefore, these results indicated that moral distress has arisen as a relevant phenomenon among healthcare workers during the COVID-19 pandemic. In Polish nursing students, Bodys-Cupak et al. studied perceived stress and sleep disorders, and found that most of the students felt high levels of stress, specifically being related to the danger of contracting COVID-19 or to a family member contagion. Regarding sleep disorders, again they were determined mainly by the fear of infection and contact with someone who may be infected with the virus, and they were closely related to the stress level. Coping strategies included denial, taking psychoactive substances, ceasing action, or blaming themselves, with higher levels of stress being related to avoidance behaviors or helplessness.

In view of the findings of this Research Topic, it seems clear that the quality of life of health workers is in danger, especially in the time of pandemic. The high rates of stress, anxiety or depression, together with other phenomena such as compassion fatigue, sleep problems or moral distress, put the health and wellbeing of healthcare workers at risk. In this sense, it is necessary to raise awareness of the psychological needs of healthcare personnel and apply prevention measures. The improvement of working conditions, the development of psychoeducational initiatives, or the strengthen of easy-accessible psychological support services, could be implemented to achieve this goal.

## Author Contributions

LG drafted the editorial. KK and NS provided opinions and suggestions on the draft. LG, KK, and NS have made a substantial and intellectual contribution to the Research Topic and have approved the editorial for publication.

## Funding

This research is part of the grant RTI2018-094089-I00, funded by MCIN/AEI/10.13039/501100011033 and FEDER Una manera de hacer Europa.

## Conflict of Interest

The authors declare that the research was conducted in the absence of any commercial or financial relationships that could be construed as a potential conflict of interest.

## Publisher's Note

All claims expressed in this article are solely those of the authors and do not necessarily represent those of their affiliated organizations, or those of the publisher, the editors and the reviewers. Any product that may be evaluated in this article, or claim that may be made by its manufacturer, is not guaranteed or endorsed by the publisher.
